# Bifactor analysis and construct validity of the five facet mindfulness questionnaire (FFMQ) in non-clinical Spanish samples

**DOI:** 10.3389/fpsyg.2015.00404

**Published:** 2015-04-09

**Authors:** Jaume Aguado, Juan V. Luciano, Ausias Cebolla, Antoni Serrano-Blanco, Joaquim Soler, Javier García-Campayo

**Affiliations:** ^1^Teaching, Research & Innovation Unit, Parc Sanitari Sant Joan de DéuSant Boi de Llobregat, Spain; ^2^Primary Care Prevention and Health Promotion Network (redIAPP)Madrid, Spain; ^3^Universitat Jaume I de CastellóCastelló, Spain; ^4^Fisiopatología de la Obesidad y la Nutrición (CIBERobn)Madrid, Spain; ^5^Department of Psychiatry, Hospital de la Santa Creu i Sant PauBarcelona, Spain; ^6^Centre for Biomedical Research in Mental HealthMadrid, Spain; ^7^Department of Psychiatry, Aragon Institute of Health Sciences (I+CS), Miguel Servet HospitalZaragoza, Spain

**Keywords:** five facet mindfulness questionnaire, bifactor model, structural equation modeling, anxiety, depression

## Abstract

The objective of the present study was to examine the dimensionality, reliability, and construct validity of the Five Facet Mindfulness Questionnaire (FFMQ) in three Spanish samples using structural equation modeling (SEM). Pooling the FFMQ data from 3 Spanish samples (*n* = 1191), we estimated the fit of two competing models (correlated five-factor vs. bifactor) via confirmatory factor analysis. The factorial invariance of the best fitting model across meditative practice was also addressed. The pattern of relationships between the FFMQ latent dimensions and anxiety, depression, and distress was analyzed using SEM. FFMQ reliability was examined by computing the omega and omega hierarchical coefficients. The bifactor model, which accounted for the covariance among FFMQ items with regard to one general factor (mindfulness) and five orthogonal factors (*observing, describing, acting with awareness, non-judgment, and non-reactivity*), fit the FFMQ structure better than the correlated five-factor model. The relationships between the latent variables and their manifest indicators were not invariant across the meditative experience. *Observing* items had significant loadings on the general mindfulness factor, but only in the meditator sub-sample. The SEM analysis revealed significant links between mindfulness and symptoms of depression and stress. When the general factor was partialled out, the *acting with awareness* facet did not show adequate reliability. The FFMQ shows a robust bifactor structure among Spanish individuals. Nevertheless, the Observing subscale does not seem to be adequate for assessing mindfulness in individuals without meditative experience.

## Introduction

According to Kabat-Zinn (1994), “*Mindfulness means paying attention in a particular way, on purpose, in the present moment, and nonjudgmentally*.” Many philosophical and religious traditions teach that happiness is found by living in the moment, and practitioners are trained to resist mind-wandering and to “be here now.” In the last decade, there has been burgeoning interest in the effectiveness of mindfulness-based therapies for a wide range of physical and mental conditions (Khoury et al., 2013). Increasing evidence supports the role of trait mindfulness and different forms of mindfulness meditation practices in enhancing psychological health and well-being in healthy and unhealthy practitioners (Keng et al., 2011; Eberth and Sedlmeier, 2012; Goyal et al., 2014).

The rapid expansion of mindfulness in different contexts has created a need to design reliable self-report measures to assess whether such practice is associated with enhanced mindfulness capacity. To date, ten different instruments, five measuring mindfulness as a general construct and five as a set of 2–5 constructs, have been developed. Despite criticism from experts in the field (Grossman, 2011), these measures are a useful way to examine the mediational mechanisms and outcomes of mindfulness-based therapies (Brown et al., 2011). Recently, Park et al. (2013) appraised and summarized the quality of ten mindfulness instruments by reviewing 46 articles that contained 79 separate studies. The authors indicated that the methodological quality of the studies included in the review was mostly good (66%) or fair (26%) across psychometric properties; however, none of the evaluated instruments are currently recommended for assessing patient-reported outcomes.

Among the published mindfulness instruments, the Five *Facet Mindfulness Questionnaire* (FFMQ; Baer et al., 2006) is considered to be the most comprehensive measure of mindfulness (Sauer et al., 2013) because it includes five distinct mindfulness components that were derived from a factor analysis of a combined item pool from five independent mindfulness instruments [Mindful Attention Awareness Scale (MAAS), Kentucky Inventory of Mindfulness (KIMS), Freiburg Mindfulness Inventory (FMI), Cognitive and Affective Mindfulness Scale Revised (CAMS-R), and the Southampton Mindfulness Questionnaire (SMQ)]. The FFMQ is a 39-item instrument with five empirically derived facets. *Observing* means noticing or attending to internal and external experiences such as sensations, thoughts, or emotions. *Describing* refers to labeling internal experiences with words. *Acting with awareness* includes focusing on one's activities in the moment as opposed to behaving mechanically. *Non-judgment of inner experience* refers to taking a non-evaluative stance toward thoughts and feelings. Finally, *non-reactivity to inner experience* is allowing thoughts and feelings to come and go, without getting caught up in or carried away by them. Four of the five facets (observing, describing, acting with awareness, and non-judgment) are identical to those present in the KIMS, a 39-item scale that was based on a concept of mindfulness as derived from Dialectical Behavior Therapy (DBT; Linehan, 1993). FFMQ items are rated on a Likert-type scale ranging from 1 (never or very rarely true) to 5 (very often or always true). The FFMQ contains both positively and negatively worded items (20 and 19, respectively), and higher scores indicate more mindfulness. Given that the relationship between the facets and the overarching construct of mindfulness differed based on meditation experience, and that associations with other constructs differed by facet, the developers recommend the use of the individual subscales instead of the total FFMQ score. According to Baer et al. (2006), a hierarchical 5-factor structure (with an overarching mindfulness factor) had the best fit in participants with meditation experience, whereas, a hierarchical 4-factor model (excluding observing) had the best fit in non-meditators. In a subsequent study, Baer et al. (2008) replicated the hierarchical 5-factor model in a sample of regular meditators. The five facets were significantly intercorrelated, but each facet explained a distinct, high proportion of variance.

Over the last decade, the psychometric properties of the FFMQ have been extensively examined in populations from different countries (Bohlmeijer et al., 2011; Deng et al., 2011; Heeren et al., 2011; Lilja et al., 2011; Cebolla et al., 2012; Sugiura et al., 2012; Dundas et al., 2013; Giovannini et al., 2014). Internal consistency coefficients of the FFMQ are adequate with Cronbach's alphas for the five facets ranging from 0.67 to 0.93 (Park et al., 2013).

The dimensionality of the FFMQ is a topic of interest among researchers. Although some authors have found a hierarchical structure in the FFMQ (e.g., Christopher et al., 2012), most recent studies suggest that a correlated 5-factor model best captures the essence of the FFMQ, as this model obtains the best fit indices in confirmatory factor analyses (CFAs). Nonetheless, the developers found that the structure of the FFMQ, particularly the Observing facet, differed between meditators and non-meditators. Bohlmeijer et al. (2011) found that the correlated 5-factor model performed slightly better than the hierarchical 5-factor model in a sample of 376 Dutch participants who had mild to moderate depressive-anxiety symptoms. de Bruin et al. (2012) indicated that the hierarchical model with five factors fit significantly worse than the non-hierarchical model in a sample of 451 university students as well as in a sample of 288 individuals who had meditation experience and were recruited from meditation centers in the Netherlands and Belgium. Van Dam et al. (2012) compared the goodness-of-fit for several potential factor configurations in the FFMQ to determine which factor structure provided the best fit in a large sample of US undergraduates. Interestingly, only the models that included “method effects” met the cut-off criteria for a “good” model. Among the tested models, the correlated 5-factor model with correlated positive and negative method factors achieved the best fit. The negative method factor accounted for more response variance than two of the original five factors. Half of the latent factor correlations were small or non-significant.

Several studies have examined the construct validity and have obtained positive correlations between the FFMQ and openness, emotional intelligence, self-compassion, and well-being and negative correlations with neuroticism, depression, anxiety, alexithymia, and dissociation (Park et al., 2013). Desrosiers et al. (2013) noted the importance of understanding the unique relationships of the five mindfulness facets with specific types of anxiety-depression symptoms and generated hypotheses about these relationships based on mindfulness and depression and anxiety theories, which were partially confirmed by a path analysis.

The present study expands upon recent research by examining, for the first time, the goodness-of-fit for a bifactor model (also known as a *nested-factor model*; Chen et al., 2006) in the FFMQ in three Spanish non-clinical samples. In a confirmatory bifactor analysis (CBFA), two types of latent factors are defined (Reise, 2012). The first latent factor is a general factor in which all items are allowed to load (representing the shared component), and the second is composed of specific factors on which items are distributed by their content. In common CBFA, all factors are mutually uncorrelated. Thus, we evaluated whether the FFMQ could be modeled with a general factor of mindfulness, as measured by 39 items, and 5 specific cognitive factors (observing, describing, acting with awareness, non-judgment, and non-reactivity), as measured by five item subsets. We expected that the bifactor model with correlated method effects would be a better fit to the data than the correlated 5-factor model with correlated method effects (Hypothesis 1), which had been the best fitting model of Van Dam et al. (2012). Our Hypothesis 1 is based on the idea that the 39 FFMQ items are multidimensional. In other words, the variance of each item can be accounted for by a single global factor (mindfulness) that reflects common variance with the other questionnaire items and by a group factor (cognitive facet) that reflects additional common variance among sets of items with highly similar content. According to Reise (2012, p. 668), the bifactor structure is very well-suited for “*representing the construct-relevant multidimensionality that arises in the responses to measures of broad constructs where multiple and distinct domains of item content are included to increase content validity*.” To evaluate the dimensionality, reliability, and validity of substantively complex measures (such as the FFMQ), a bifactor structural model may be an excellent alternative to the more commonly-tested correlated or second-order (hierarchical) representations of an instrument's latent structure. According to Chen et al. (2006), bifactor models have some advantages compared to hierarchical models. One of the most important advantages is that in the bifactor model, the relationship of the group (or specific) factors to prediction of an external variable can be studied independently of the general factor. This is crucial for our subsequent construct validity analysis, in which we want to know the relationship between the FFMQ cognitive facets and the DASS-21 latent factors (partialling out the influence of the FFMQ general factor). Another important advantage is that the bifactor model allow researchers to examine measurement invariance at both the general and group factor levels. In the hierarchical model, measurement invariance is studied at the general factor level only. In addition, in the bifactor model, group mean differences can be studied at both general and group factor levels. Second, we tested invariance of our best-fitting model's parameter estimates across distinct meditative experience (regular meditators vs. non-meditators), following a sequence of different restrictive constraints based on theoretical assumptions. Specifically, we expected that the method factors, the general mindfulness factor, and the Observing facet were not invariant across meditative practice (Hypothesis 2). Third, CBFA also helps evaluate whether the computation of factor scores is justifiable or whether only the total score should be computed and reported. In this case, we evaluated the reliability of the five facet scores beyond the reliability provided by the general factor (mindfulness). According to Van Dam et al. (2012), “*the lack of a superordinate mindfulness factor in the present analyses suggests that the subscales may be related but cannot be considered as being subsumed (at least statistically) by a hierarchical factor*.” Thus, we expect that the subscales' capacity to reliably measure the variance due to the specific factors is considerably high (Hypothesis 3). Finally, taking Desrosiers et al. (2013) theoretical framework into account, we examined relationships for each of the latent FFMQ factors with anxiety, depression, and distress symptoms (DASS-21) through structural equation modeling (SEM). Specifically, we expected that the latent factor of *observing* would contribute to heightened introspective awareness, and thereby to anxiety (Hypothesis 4), *describing* would be associated with depression (Hypothesis 5), *acting with awareness* would be associated with general distress (Hypothesis 6), *non-judgment* would be associated with both depression and general distress (Hypothesis 7), and *non-reactivity* would be associated with general distress (Hypothesis 8).

## Method

### Settings and samples

In the present work we used the datasets from three observational, cross-sectional studies. All were approved by local Ethics Committees and performed in accord with the ethical standards of the 1964 Declaration of Helsinki.

In the first study (Cebolla et al., 2012), 279 undergraduate and postgraduate students enrolled in Psychology studies at the Spanish Universities of Valencia and Castellón completed a paper-and-pencil battery of instruments in a classroom. Additionally, 54 participants recruited from the general population completed the measures. Therefore, a total of 333 adult individuals participated in the study (Sample 1). They did not receive remuneration or academic incentives. All were informed that their answers were confidential and signed an informed consent before completing the study measures.

In the second study (Soler et al., 2014), a survey containing a battery of instruments was developed using a commercial online system (www.surveymonkey.com; Portland, OR, USA). The survey was available for response between April 2011 and December 2012. A total of 688 adult individuals completed the survey (Sample 2). Participants accessed a link to the online study that was posted on several Spanish scientific research portals involved in mindfulness and meditation research. In addition, members from several mindfulness associations, Zen monasteries, and Sanghas were provided a link to the online study. Finally, a non-meditator convenience sample completed the online survey.

The third group of participants (Sample 3) were 173 undergraduates, enrolled in Psychology studies at the University of Castellón, Spain, who completed a battery of instruments (including the FFMQ). They did not receive remuneration or academic incentives for their participation in this study.

### Measures

Participants completed a socio-demographic questionnaire, meditation questions, the DASS-21, and the FFMQ as part of a battery of instruments administered online or by paper-and-pencil.

#### Meditation experience

All participants from Sample 2 were asked about meditation practice using the following question: *“have you ever practiced any kind of meditation?”* If participants responded “*yes*,” then they were instructed to answer additional questions, including, *“What kind of meditation?”; “How long have you been practicing meditation?”; “how often do you practice per week?”;* and *“how long do you practice in each session?”*

#### The depression anxiety stress scales-21 (DASS-21; Lovibond and Lovibond, 1995; Bados et al., 2005)

We had DASS-21 data only on individuals from Sample 2 (*n* = 688). The DASS-21 is a self-report instrument that was developed to distinguish between features of depression (low positive affect), anxiety (physical arousal), and stress (psychological tension/agitation) in clinical and non-clinical samples. The respondent is required to indicate the presence of a symptom over the previous week. Each item is scored from 0 (*did not apply to me at all over the last week*) to 3 (*applied to me very much or most of the time over the past week*). There are seven items on each of the three DASS sub-scales (Depression, Anxiety, and Stress). Therefore, total scores in each scale can range from 0 to 21. The DASS-21 showed a clear bifactor structure (one general distress/negative affect dimension plus three domain-specific dimensions) in a sample of North-American undergraduates (Osman et al., 2012), which corroborated previous results reported by Henry and Crawford (2005) in large, non-clinical adult samples from the UK. Bados et al. (2005) examined the psychometric properties of the Spanish version of the DASS-21 and found that the three-factor model provided the best fit in a sample of 365 Spanish university students. The analyses revealed adequate internal consistency for each subscale (Depression = 0.84; Anxiety = 0.70, and Stress = 0.82), satisfactory convergent validity and modest discriminant validity. These authors did not test the goodness-of-fit of a bifactor model in the Spanish DASS-21.

#### The five facet mindfulness questionnaire (FFMQ; Baer et al., 2006; Cebolla et al., 2012)

Cebolla et al. (2012) assessed the psychometric properties of the Spanish version of the FFMQ in Sample 1 (described above) and in 146 patients with different mental disorders (borderline personality disorder, major depression, eating disorders, etc.), who were recruited from two mental health units in Valencia and Barcelona, Spain. The FFMQ was initially translated into Spanish by a group of mindfulness experts. Then, a bilingual psychologist from the USA performed back translation. Discrepancies with the original English version were resolved by a professional English translator. The authors contrasted three different factor models using CFA (the bifactor model was not contrasted) and concluded that the hierarchical 4-factor model solution (with the Observing facet isolated) best represented the data. One of the principal shortcomings of this study was the use of item parcels. Tran et al. (2013) recently noted that item parcels may yield spuriously high model fit and model misspecifications. The five mindfulness facets were reliable (all α = 0.80). Overall, the patterns of correlations with the psychopathology, mindfulness, and acceptance instruments were in the expected directions.

Socio-demographic data, meditation experiences, FFMQ facet scores, and DASS-21 sub-scale scores from each study sample are shown in Tables 1, 2.

**Table 1:** **Participant characteristics for the three samples**.

**Characteristics**	**Sample 1 (*N* = 333)**	**Sample 2 (*N* = 688)**	**Sample 3 (*N* = 173)**
Gender (female): n (%)	123 (38.7)	428 (62.2)	NA
Age (years): M (SD)	26.5 (8.73)	41.4 (11.1)	22.5 (4.6)
Level of education: n (%)			
Primary school	NA	13 (1.9)	0
Secondary school	NA	133 (19.3)	0
University	NA	542 (78.8)	173 (100)
FFMQ (M, SD)			
Observing	23.5 (5.3)	28.4 (5.5)	23.2 (5.3)
Describing	30.2 (5.5)	29.9 (5.7)	28.7 (5.4)
Acting with Awareness	28.2 (6.0)	26.8 (5.5)	27.9 (6.0)
Non-Judgment	28.7 (6.4)	29.3 (6.8)	28.6 (6.4)
Non-Reactivity	21.5 (4.2)	23.3 (4.6)	20.1 (4.0)
DASS-21 (M, SD)			
Depression	NA	10.6 (4.2)	NA
Anxiety	NA	9.9 (3.5)	NA
Stress	NA	12.9 (3.9)	NA
Meditation experience			
Yes (>8 weeks)	NA	305 (44.3)	NA
No	NA	287 (41.7)	NA
Missing	NA	96 (14.0)	NA

*NA: not available*.

**Table 2:** **Characteristics of meditation practices in meditators (*N* = 305)**.

**Characteristics**	**Meditators (*N* = 305)**
Years meditated: *n* (%)	
Less than 1 year	33 (10.8)
1–5 years	144 (47.2)
6–10 years	66 (21.6)
≥11 years	62 (20.3)
Meditation hours per week: *n* (%)	
<1 h	101 (33.1)
1–3 h	147 (48.2)
4-6 h	39 (12.8)
≥7 h	18 (5.9)
Type of meditation: *n* (%)	
Mindfulness	105 (34.4)
Zen	81 (26.6)
Yoga	22 (7.2)
Other	11 (3.6)
Missing	86 (28.2)

### Statistical analyses

SAS 9.3 and MPlus 7.12 (Muthén and Muthén, 1998–2012) were used to conduct the data analyses.

#### Factor analyses

**Figure 1:**
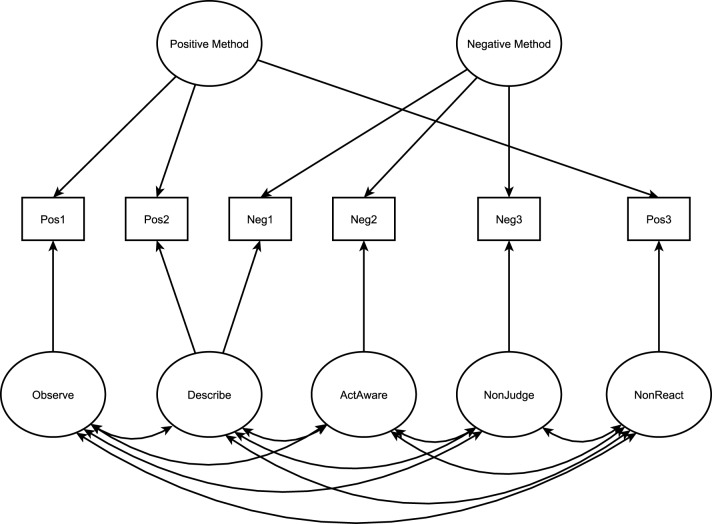
**Model tested for the FFMQ: Correlated 5-factor model + uncorrelated negative and positive method factors**. Individual items have been grouped for illustrative purposes. Analysis performed with individual items.

**Figure 2:**
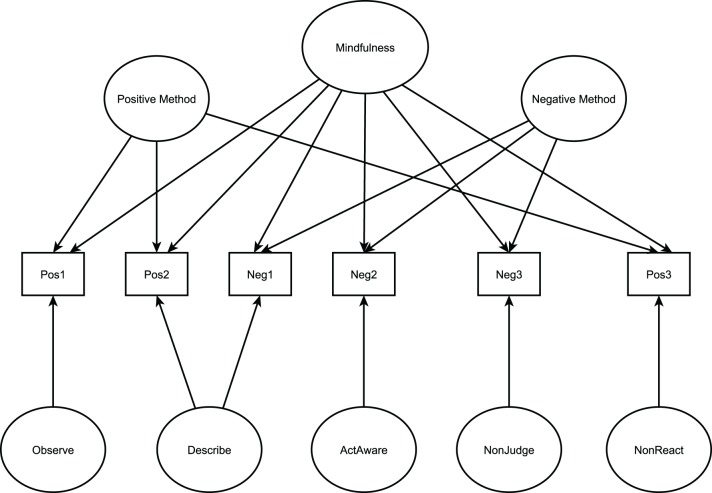
**Model tested for the FFMQ: Bifactor model + uncorrelated negative and positive method factors**. Individual items have been grouped for illustrative purposes. Analysis performed with individual items.

The factor structure of the FFMQ and the DASS-21 (the instrument used for the construct validity analysis) were evaluated with CFAs. Participants with missing values on all FFMQ and DASS-21 items were excluded from the analyses. For the FFMQ, two models were tested in the pooled sample^1^ (*N* = 1191; see Figures 1, 2): (a) a correlated 5-factor model with positive and negative method factors as proposed by Van Dam et al. (2012) and (b) a bifactor model positing that all items load on a general latent factor of mindfulness and on five specific uncorrelated cognitive facets. Positive and negative method factors were included in this model. In bifactor models, interpretations of facet scores for specific constructs should emphasize that these scores represent the joint functioning of both general (mindfulness) and specific (cognitive) factors. We considered items with moderate to high standardized facet loadings (values ≥ 0.30) to be strongly linked with its corresponding specified facet.

The fit of the CBFA model proposed by Osman et al. (2012) for the DASS-21 was tested in sample 2 (*N* = 679). This model defines a general distress factor and three specific, uncorrelated factors: depression, anxiety, and stress. To assess the fit of the factor models the following fit indices were used; the chi-square with degrees of freedom, Comparative Fit Index (CFI), root-mean-square error of approximation (RMSEA), standardized root-mean-square residual (SRMR), Akaike information criterion (AIC) and Bayesian information criterion (BIC). Given that fit indices are influenced by data distributions, model parameters, and sample size, we present both conservative and liberal cut-offs for an acceptable fit (Schermelleh-Engel et al., 2003). A model that adequately fits the data will have: CFI ≥ 0.95 (conservative) or ≥ 0.90 (liberal), RMSEA ≤ 0.06 (conservative) or ≤ 0.10 (liberal), SRMR ≤ 0.05 (conservative) or ≤0.10 (liberal). The AIC and BIC have no specific value that suggests a “good” model fit but are helpful when comparing non-nested models as lower values indicate better fit.

#### Factorial invariance of the FFMQ

Multigroup CFA was employed to test the invariance of parameter estimates between regular meditators and non-meditators. The focus was on two levels of invariance (Byrne, 2012): (1) configural invariance, in which the best fitting model found in the CFA of the FFMQ was tested in a multigroup framework and no equality constraints were imposed on the parameters between groups. A satisfactory fit in this model implies that the number of factors and the factor loading pattern are the same across groups; and (2) measurement invariance, which examined the factor loadings and their equality across meditation groups. Based on findings from previous research, we examined the invariance of factor loadings in the following components of the model; (i) the Observing facet (Van Dam et al., 2012; Williams et al., 2014), (ii) the method factors (Van Dam et al., 2012) and (iii) the general mindfulness factor. A sequence of constrained models nested to the configural model were defined to establish invariance in the three aforementioned components. Differences in CFI, RMSEA and SRMR together with the result of the Likelihood Ratio Test (LRT) after a Bonferroni adjustment (Sass, 2011) assessed if the goodness-of-fit of the model was significantly worse after constraints were introduced. It is known that the LRT is too sensitive to sample size and that it should be complemented with other criteria (Chen, 2007; Meade et al., 2008; Sass, 2011). Based on research by Chen (2007), acceptable model fit for more restrictive invariant models are as follows: ΔCFI < 0.01, ΔRMSEA < 0.015 and ΔSRMR < 0.03. These analyses were conducted on sample 2 for participants with non-missing meditation data (*N* = 592).

#### Reliability estimates

Two types of reliability indices can be computed for CBFA models: Omega (ω) and omega hierarchical (ω-h) (Brunner et al., 2012). ω is the reliability of a summed score formed with all the factors that comprise that score. ω-h, which can be equal to or smaller than ω, is the reliability of a summed score that consists of only one construct. For the general factor (mindfulness), the difference between ω and ω-*h* provides information about the reliability of the total score resulting from its specific factors. For the specific factors, ω-*h* provides information about the capacity of the subscale scores to reliably measure the variance attributable to the specific factors by themselves, partialling out the reliability provided by the general factor. Low ω-*h* values deter the use of subscale scores. Two important statistical requirements need to be fulfilled for computing ω and ω-*h*: (1) the target model has to fit the observed data well, and (2) parameter estimates need to be precise.

#### Construct validity analysis

The relationship between the latent factors of the FFMQ and the DASS-21 were examined with a SEM analysis using the best fitting model of the FFMQ and the bifactor model of the DASS-21. Following Desrosiers et al. (2013) framework, each DASS-21 latent factor was simultaneously regressed onto the FFMQ factors, controlling for intercorrelations. Sample 2 was used after excluding participants who had missing values on all items of the FFMQ or DASS-21 (*N* = 679).

The parameters for the CFAs and the SEM model were estimated using the Robust Maximum Likelihood (MLR) method. This method has been selected over a weighted least square mean and variance estimator (WLMV) despite the categorical nature of the data for two main reasons: (i) all items had at least 4 categories (Beauducel and Herzberg, 2006); (ii) the MPlus implementation of MLR produces correct results even in the presence of data missing at random (Muthén and Muthén, 1998–2012), which is not the case for WLSMV. A robust version of the ML estimator was selected to account for the skewness in the distribution of the DASS-21 items.

## Results

### Descriptive statistics

Descriptive statistics for the FFMQ items are presented in Table 3.

**Table 3:** **Mean (M), standard deviation (SD), and standardised factor loadings (λ) for the FFMQ bifactor model with uncorrelated method effects**.

**Items**	**M (SD)**	**λ*gen***	**λ specific**	**Method factor (neg)**	**Method factor (pos)**
**OBSERVING**					
1. When I'm walking, I deliberately notice the sensations of my body moving.	2.80 (1.10)	*0.01*	0.46		0.49
6. When I take a shower or bath, I stay alert to the sensations of water on my body.	3.18 (1.13)	*0.04*	0.49		0.46
11. I notice how foods and drinks affect my thoughts, bodily sensations, and emotions.	2.65 (1.28)	−0.12	0.38		0.46
15. I pay attention to sensations, such as the wind in my hair or sun on my face.	3.31 (1.14)	*0.05/*	0.64		0.49
20. I pay attention to sounds, such as clocks ticking, birds chirping, or cars passing.	3.32 (1.04)	*0.02*	0.53		0.37
26. I notice the smells and aromas of things.	3.81 (0.94)	0.15	0.39		0.41
31. I notice visual elements in art or nature, such as colors, shapes, textures, or patterns of light and shadow	3.54 (1.14)	*0.04*	0.41		0.47
36. I pay attention to how my emotions affect my thoughts and behavior.	3.66 (0.92)	*0.01*	0.18		0.48
**DESCRIBING**					
2. I'm good at finding words to describe my feelings.	3.71 (0.88)	0.27	0.69		0.32
7. I can easily put my beliefs, opinions, and expectations into words.	3.82 (0.87)	0.28	0.65		0.35
12. It's hard for me to find the words to describe what I'm thinking.	2.19 (0.93)	0.45	0.67	*0.07*	
16. I have trouble thinking of the right words to express how I feel about things.	2.10 (0.87)	0.53	0.63	0.09	
22. When I have a sensation in my body, it's difficult for me to describe it because I can't find the right words.	2.16 (0.83)	0.51	0.45	*−0.04*	
27. Even when I'm feeling terribly upset, I can find a way to put it into words.	3.67 (0.93)	0.34	0.58		0.30
32. My natural tendency is to put my experiences into words.	3.44 (1.04)	*0.02*	0.62		0.30
37. I can usually describe how I feel at the moment in considerable detail.	3.63 (0.95)	0.27	0.70		0.32
**ACTING WITH AWARENESS**					
5. When I do things, my mind wanders off and I'm easily distracted.	2.91 (0.99)	0.44	0.52	0.46	
8. I don't pay attention to what I'm doing because I'm daydreaming, worrying, or otherwise distracted.	2.47 (0.94)	0.51	0.50	*0.23*	
13. I am easily distracted.	2.79 (0.99)	0.50	0.54	0.49	
18. I find it difficult to stay focused on what's happening in the present.	2.39 (0.93)	0.61	0.39	*0.14*	
23. It seems I am “running on automatic” without much awareness of what I'm doing.	2.38 (1.03)	0.33	0.45	*−0.13*	
28. I rush through activities without being really attentive to them.	2.47 (0.91)	0.54	0.51	*−0.11*	
34. I do jobs or tasks automatically without being aware of what I'm doing.	2.64 (0.91)	0.46	0.70	*−0.20*	
38. I find myself doing things without paying attention.	2.64 (0.91)	0.44	0.67	*−0.14*	
**NON-JUDGMENT**					
3. I criticize myself for having irrational or inappropriate emotions.	2.80 (1.09)	0.47	0.53	*0.05*	
10. I tell myself I shouldn't be feeling the way I'm feeling.	2.36 (1.01)	0.55	0.54	*0.01*	
14. I believe some of my thoughts are abnormal or bad and I shouldn't think that way.	1.99 (1.02)	0.64	0.48	*0.01*	
17. I make judgments about whether my thoughts are good or bad.	2.66 (1.09)	0.53	0.51	*−0.02*	
25. I tell myself that I shouldn't be thinking the way I'm thinking.	2.20 (1.01)	0.57	0.61	*−0.09*	
30. I think some of my emotions are bad or inappropriate and I shouldn't feel them.	2.08 (0.98)	0.60	0.57	*−0.11*	
35. When I have distressing thoughts or images, I judge myself as good or bad, depending what the thought/image is about.	2.32 (1.03)	0.53	0.45	*−0.05*	
39. I disapprove of myself when I have irrational ideas.	2.56 (1.14)	0.45	0.62	*−0.04*	
**NON-REACTIVITY**					
4. I perceive my feelings and emotions without having to react to them.	3.16 (0.94)	0.23	*0.08*		0.52
9. I watch my feelings without getting lost in them.	3.14 (0.91)	0.31	*0.03*		0.50
19. When I have distressing thoughts or images, I “step back” and am aware of the thought or image without getting taken over by it.	3.30 (1.00)	0.25	0.34		0.53
21. In difficult situations, I can pause without immediately reacting.	3.16 (0.93)	0.28	*0.06*		0.51
24. When I have distressing thoughts or images, I feel calm soon after.	3.42 (0.96)	0.41	0.54		0.34
29. When I have distressing thoughts or images I am able just to notice them without reacting.	2.94 (0.95)	0.16	0.28		0.57
33. When I have distressing thoughts or images, I just notice them and let them go.	3.18 (0.95)	0.33	0.56		0.47

*Non-significant loadings in italics*.

### Dimensionality analyses

Fit indices for the tested FFMQ models are presented in Table 4. In support of Hypothesis 1, the bifactor model with correlated method effects (M1) was a better fit to the data than the correlated 5-factor model with correlated method effects (M2), as fit indices indicated good fit (CFI > 0.95, RMSEA and SRMR < 0.05). Unexpectedly, the correlation between the positive and negative method factors was not statistically significant in any of the models (*p* = 0.99 and 0.88 for M1 and M2, respectively), consequently, both models were re-estimated with the correlation fixed to zero (models M3 and M4). The bifactor model with uncorrelated method effects (M3) was a better fit to the data than the correlated factor model with uncorrelated method effects (M4).

**Table 4:** **Fit statistics for the FFMQ and DASS-21 latent structure models**.

**Model**	**MLRχ^2^ (df)**	**CFI**	**RMSEA (90% CI)**	**SRMR**	**AIC**	**BIC**
**FFMQ (Pooled sample, *N* = 1191)**						
M1 Bifactor model + correlated method effects	1432.63 (623)	0.955	0.033 (0.031;0.035)	0.035	108472.8	109469.0
M2 Correlated 5-factor model + correlated method effects	1708.6 (652)	0.942	0.037 (0.035;0.039)	0.048	108767.8	109616.6
M3 Bifactor model + uncorrelated method effects	1431.87 (624)	0.956	0.033 (0.031;0.035)	0.035	108470.8	109461.9
M4 Correlated 5-factor model + uncorrelated method effects	1706.2 (653)	0.942	0.037 (0.035;0.039)	0.048	108765.9	109609.5
**DASS-21 (Sample 2, *N* = 679)**						
Bifactor model	368.10 (168)	0.963	0.042 (0.036;0.048)	0.030	24462.7	24842.4
**Construct Validity (Sample 2, *N* = 679)**						
FFMQ (M3) & DASS-21	2537.99 (1587)	0.951	0.030 (0.028;0.032)	0.037	81767.2	83136.9

The standardized factor loadings for the best-fitting model (M3) are displayed in Table 3. For the general factor, loading values ranged from small to large (*M* = 0.34, range = 0.01-0.64) and were considerably varied among items for the different facets. The items with the lowest loadings on the general factor were from the *Observing* facet (*M* = 0.06, range = 0.01–0.15), with only two items reaching statistical significance, while the *Non-judgment* facet had loadings that were moderate to large (*M* = 0.54, range = 0.45-0.64). For the specific factors, loadings were, in general, higher (or equal) than the loadings on the general factor. The specific factor with the lowest loadings was *Non-reactivity* (*M* = 0.27, range = 0.03–0.56), whereas the *Describing* factor had the highest loadings (*M* = 0.62, range = 0.45–0.70). It should be highlighted that the *Observing* specific factor had loadings that were all statistically significant and, with the exception of item 36, higher than 0.30 (*M* = 0.44, range = 0.18-0.64), which was in contrast to the aforementioned *Observing* item loadings onto the general factor. Finally, it is noteworthy that most factor loadings on the negative method factor were small (*M* = 0.13, range = 0.01-0.49) and not statistically significant, whereas the positive items were strongly linked to the corresponding method factor (14 of 20 items with loading values ≥ 0.40; *M* = 0.43, range = 0.30-0.57).

### Measurement invariance of the best-fitting FFMQ model across meditative experience (meditators vs. non-meditators)

The baseline model (Model 1) requires that the same item be an indicator for the same latent factor in each group, while factor loadings can vary across groups. This model had an acceptable fit (Table 5), indicating that the configural invariance of the FFMQ holds across meditative experience. Next, factor loadings were constrained to be equal in the two groups to test for weak invariance (Model 2). The fit of this model compared to the baseline model was significantly worse as indicated by the likelihood ratio test (*p* < 0.001) and CFI (ΔCFI ≥ 0.01) and SRMR (ΔSRMR ≥ 0.03). These results suggest that some loadings are different between the two groups while the factor structure remains the same. We focused on specific hypotheses derived from previous studies to identify which loadings are the source of the invariance (Van Dam et al., 2012; Williams et al., 2014). In Model 3, the factor loadings of the *observing* items on the general factor were not constrained to be equal between groups. The fit of Model 3 was not worse than the Model 1 according to the ΔCFI, ΔSRMR, and ΔRMSEA, whereas the LRT indicated a significant difference in the chi-square (*p* < 0.001). From a conservative approach, because the LRT is sensitive to sample size (Chen, 2007), we conclude that a model with different loadings for the *observing* items on the general factor and equal loadings for the specific factor is parsimoniously preferable over the baseline model (unconstrained). Because the LRT was still indicating significant differences with the baseline model, we followed the course of the more constrained models that were in agreement with our initial hypotheses. According to the LRT, Model 6 was not worse than the baseline model. Thus, we chose the model with all the loadings equal between groups except for the ones related to *observing*, method effects, and general mindfulness. This result is in support of Hypothesis 2.

**Table 5:** **Measurement invariance of the FFMQ bifactor model across meditative experience (meditators vs. non-meditators^2^)**.

**Model**	**MLRχ^2^ (df)**	**CFI**	**RMSEA**	**SRMR**	**Model Comparison**	**ΔMLRχ^2^ (Δdf)**	**ΔCFI**	**ΔRMSEA**	**ΔSRMR**
1. Baseline model (no constraints)	1791.28 (1246)	0.947	0.038	0.046	–	–			
2. All factor loadings invariant (measurement invariance)	2022.70 (1363)	0.936	0.040	0.079	2 vs. 1	214.62^*^ (117)	0.011†	0.002	0.033†
3. All factor loadings invariant except those for observe on the general mindfulness factor	1978.38 (1355)	0.939	0.040	0.074	3 vs. 1	185.43^*^ (109)	0.008	0.002	0.028
4. All factors loadings invariant except all those related to observe	1943.18 (1339)	0.941	0.039	0.064	4 vs. 1	146.59^*^ (93)	0.006	0.001	0.018
5. All factors loadings invariant except those related to observe and wording effects	1892.54 (1308)	0.943	0.039	0.060	5 vs. 1	98.32^*^ (62)	0.004	0.001	0.014
6. All factors loadings invariant except observe, wording effects, and general mindfulness	1821.04 (1277)	0.947	0.038	0.051	6 vs. 1	34.82 (31)	0	0	0.005

* *Significant after Bonferroni adjustment*.

† *ΔCFI ≥ 0.01; ΔRMSEA ≥ 0.015; ΔSRMR ≥ 0.03*.

In sum, using the de Bruin et al. (2012) terminology, the FFMQ has “configural invariance” because the same factor structure holds across groups (meditators and non-meditators) but not “metric invariance” because the factor loadings differ across groups. Favoring the combined use of the LRT and the difference in fit indices, we conclude that the factor loadings for the observing items on the general factor of mindfulness are responsible for the main differences between meditators and non-meditators. Thus, the observing items showed higher loadings on mindfulness in meditators (*M* = 0.29, range = 0.18–0.37) than in non-meditators (unsigned *M* = 0.09, range= −0.27−0.13). Remarkably, only item 11 loaded significantly (−0.27) on mindfulness in the non-meditators group.

### Reliability

In our study, ω values reflect how well each specific factor score measures the blend of mindfulness and the corresponding specific facets. The ω values of 0.85 (observing),0.90 (describing),0.91 (acting with awareness), 0.92 (non-judgment), and 0.82 (non-reactivity), indicate that 85, 90, 91, 92, and 82% of the variance, respectively, is attributable to the blend of mindfulness and the specific facet being measured. Relatedly, ω-*h* coefficients estimate the proportion of variance in raw scores attributable to a single specific facet. The ω-*h* values of 0.84 (observing), 0.78 (describing), 0.01 (acting with awareness), 0.72 (non-judgment), and 0.76 (non-reactivity) indicate that 84, 78, 1, 72, and 76% of the variance, respectively, is attributable to each specific facet. In other words, with the exception of acting with awareness, when the general mindfulness factor is controlled, the facets reliably measure the variance due to each specific facet, which supports Hypothesis 3. Finally, the ω and ω-*h* estimates for the total FFMQ score were 0.93 and 0.53, respectively. The difference between ω and ω-*h* suggests that the specific facets have considerable influence on the reliability of the FFMQ total score. This finding provides additional support for using multidimensional scoring procedures as the scores obtained from the 39 FFMQ items are not reflective of a single common source.

### Construct validity analysis using Desrosiers et al. (2013) theoretical model as a framework

As shown in Table 4, the bifactor model of the DASS-21 proposed by Osman et al. (2012) demonstrated a good fit to the data. The item loadings on the general distress factor were all above 0.30 and were statistically significant (*M* = 0.64, range = 0.39-0.75). For the specific factors, only the *depression* loadings were moderate to large (*M* = 0.41, range = 0.29-0.62) and were significant. In contrast, several *anxiety* and *stress* loadings were not statistically significant (Items 2, 4, and 19 for *anxiety* and items 6, 11, 14, and 18 for *stress*) with factor loadings within the -0.10–0.35 range (unsigned *M* = 0.23) for *anxiety* and 0.05–0.50 (*M* = 0.23) for *stress*.

SEM analysis assessed the construct validity of the FFMQ bifactor structure. As shown in Table 4, all fit indices were within conservative acceptable limits (CFI ≥ 0.95, RMSEA ≤ 0.06, and SRMR ≤ 0.05). The results of each DASS-21 latent factor regressed simultaneously onto the FFMQ factors are presented in Table 6. Contrary to Hypothesis 4, we found a close to significant negative relationship between *observing* and *anxiety* (*p* = 0.052). The other expected relationships (Hypotheses 5-8) did not receive empirical support. We found a significant negative effect of the general mindfulness factor on *depression* (*p* = 0.04), *stress* (*p* = 0.03) and *general distress* (*p* < 0.001). In addition, only *observing* and *stress* were significantly related (*p* = 0.007).

**Table 6:** **Standard estimates for FFMQ factors regressed simultaneously on the specific (Depression, Anxiety and Stress) and general factors of the DASS-21**.

**Estimate (95%CI)**	**DASS-21 factors**			
	**Depression**	**Anxiety**	**Stress**	**General distress**
**Bifactor FFMQ model**				
Observing	−0.12 (−0.27;0.03)	−0.28 (−0.56;0.00)†	−0.23 (−0.39; −0.06)^**^	0.06 (−0.07;0.19)
Describing	−0.10 (−0.27;0.07)	−0.04 (−0.33;0.26)	0.09 (−0.07;0.25)	−0.12 (−0.24;0.00)†
Acting with awareness	−0.09 (−0.24;0.06)	−0.02 (−0.32;0.29)	−0.21 (−0.42;0.01)†	−0.05 (−0.18;0.07)
Non-judgment	−0.14 (−0.35;0.08)	−0.18 (−0.59;0.24)	−0.16 (−0.38;0.05)	−0.08 (−0.24;0.07)
Non-reactivity	−0.10 (−0.33;0.12)	−0.22 (−0.59;0.16)	−0.30 (−0.62;0.01)†	−0.08 (−0.24;0.09)
Mindfulness	−0.26 (−0.51; −0.01)^*^	−0.15 (−0.60;0.32)	−0.41 (−0.78; −0.04)^*^	−0.66 (−0.79; −0.53)^**^
*R*^2^	0.13	0.18	0.39	0.47
**Correlated FFMQ model**				
Observing	−0.12 (−0.27;0.04)	−0.30 (−0.59; −0.01)^*^	−0.21 (−0.41; −0.01)^*^	0.08 (−0.06;0.21)
Describing	−0.11 (−0.28;0.06)	−0.03 (−0.37;0.31)	0.11 (−0.06;0.27)	−0.21 (−0.33; −0.08)^**^
Acting with awareness	−0.06 (−0.21;0.08)	0.08 (−0.14;0.31)	−0.19 (−0.36; −0.02)^*^	−0.16 (−0.32;0.00)†
Non-judgment	−0.12 (−0.30;0.06)	−0.13 (−0.47;0.21)	−0.13 (−0.32;0.06)	−0.24 (−0.40; −0.09)^**^
Non-reactivity	−0.07 (−0.27;0.13)	−0.04 (−0.41;0.34)	−0.30 (−0.49; −0.10)^**^	−0.27 (−0.44; −0.09)^**^
*R*^2^	0.13	0.13	0.38	0.44

† *p < 0.10*.

* *p < 0.05*.

** *p < 0.01; 95%CI = 95% Confidence Interval*.

In a supplementary analysis, we studied the construct validity of the FFMQ without disentangling mindfulness from its facets. In other words, we posited that the general factor of mindfulness accounts for the significant link between cognitive facets and depression/anxiety that has been reported in previous works. When mindfulness was integrated with its facets, additional significant relationships flourished. The expected negative relationship between *acting with awareness* and general distress was supported (Hypothesis 6), although it was only marginally significant. Additionally, *non-judgment* (*p* = 0.002) and *non-reactivity* (*p* = 0.003) were significantly associated with general distress, providing partial support for Hypothesis 7 and total support for Hypothesis 8. The effect of *observing* on *anxiety* was statistically significant, but again it was not in the expected positive direction. None of the expected effects on the depression factor (Hypothesis 5 for *describing* and Hypothesis 7 for *non-judgment*) emerged in this model.

## Discussion

The FFMQ is one of the most frequently used assessment instruments in mindfulness research (Park et al., 2013; Sauer et al., 2013); however, several psychometric aspects have remained unexplored. For example, consistent with Baer et al's (2006) seminal work, the underlying dimensionality of the questionnaire was examined in prior research, without testing the goodness-of-fit of a bifactor structure. We explicitly compared two alternative models of the FFMQ measurement structure in non-clinical Spanish individuals to address this research gap: a bifactor model with method effects *versus* the correlated 5-factor model with method effects (Van Dam et al., 2012). Although both models met the cut-off criteria for good model fit, the CFAs provided more empirical support for a bifactor structure. Consistent with Hypothesis 1, the CFA indicated that the FFMQ consisted of a global mindfulness factor and five specific cognitive facets. With the exception of most observing items and one describing item (item 32), the FFMQ items consistently loaded on both the general mindfulness factor and the corresponding domain-specific facet, which indicates that the FFMQ items should be conceptualized as multidimensional rather than specific. Given the identified bifactor or nested-factor structure in the FFMQ, clinicians and researchers should consider that the FFMQ facets scores represent the joint functioning of a general factor (mindfulness) and specific constructs (cognitive facets). Because most item loadings on the specific factors were high (34 of 39 items >0.30), we conclude that the specific factors are well defined even in the simultaneous presence of the global factor of mindfulness.

The FFMQ contains both positively worded (mindfulness capacity) and negatively worded (mindfulness deficit) items. Höfling et al. (2011) and Van Dam et al. (2012) have highlighted the importance of taking method effects into account when mindfulness is measured with both positively and negatively worded items. Specifically, Van Dam et al. (2012) reported that only the factor models that included method effects met the cut-off criteria for a good model. Their analyses of FFMQ data in a large sample of US undergraduates showed that a correlated 5-factor model with two correlated method factors, one capturing negatively worded items and one capturing positively worded items, fit the data significantly better than the same model without method factors, with only one method factor, or with uncorrelated method factors. In contrast with Van Dam et al. (2012), our results found an orthogonal relationship between the positive and negative method factors. The absence of a significant correlation between positive and negative method factors may suggest that Spanish individuals susceptible to negative method effects are not simultaneously susceptible to positive method effects and *vice versa*. It is also important to note that positively worded items had moderate standardized loadings on the corresponding method factor, whereas the negatively worded items did not. These findings are in line with those reported by Höfling et al. (2011), who examined a modified version of the Mindful Attention and Awareness Scale (MAAS). The authors observed that the factor loadings for the negatively worded items were lower than the loadings for the positively worded items (0.28–71 vs.0.31–0.78, respectively).

Because a lack of factorial invariance can undermine valid score interpretations, it was crucial to examine whether certain aspects of the FFMQ are not comparable across groups of respondents with different meditation experience. Therefore, as a second step, we tested the factor structure invariance across meditators and non-meditators (multigroup CFA of the bifactor model). The results from the invariance analyses showed that the same factor configuration holds across groups (configural model), but the pattern of factor loadings was not identical across groups. Altogether, our results suggest that two FFMQ versions should be differentiated in the context of applied clinical research: one with five facets (39 items) for meditators and another with four facets (31 items; excluding observing) for non-meditators. This finding supported Hypothesis 2 and was also consistent with findings from Williams et al. (2014), who recently indicated that it is important to include only facets that empirical evidence suggests are key facets of mindfulness when comparing meditators and non-meditators. They posit that including the observing items when examining mindfulness scores in non-meditator adult samples may result in biased scores because observing may mean something different to meditators and non-meditators.

To date, all studies had computed Cronbach alphas (α) to estimate the reliability of the FFMQ facet and total scores (e.g., Bohlmeijer et al., 2011; de Bruin et al., 2012). There are two reliability statistics, coefficients ω and ω-*h*, which can be computed with bifactor models. Of special interest is the ω-*h* statistic, which allows for an examination of the degree to which the FFMQ represents a multidimensional construct, or a single, global mindfulness factor. Furthermore, ω-*h* is a less biased estimate (compared to Cronbach's α) of the reliability of facet scores (Reise, 2012). Overall, our reliability analyses recommend against using the FFMQ total score, whose ω-*h* value was modest. In their recent review, Park et al. (2013) also indicated a preference for using the individual subscales instead of the total FFMQ score. Supporting Hypothesis 3, FFMQ facet scores showed relatively high reliability (in terms of ω-*h*) in assessing specific cognitive facets because they did not contain a large amount of variance attributable to the global factor. The exception was the facet, acting with awareness, whose ω and ω-*h* estimates were 0.91 and 0.01, respectively. The difference between ω and ω-*h* suggests that mindfulness has a large influence on the reliability of this facet, which supports that the scores obtained from this facet result from a single common source that should not be scored using multidimensional scoring procedures. Thus, the representativeness of this specific cognitive dimension remains questionable because it does not appear to represent a legitimate construct. In contrast to acting with awareness, the observing facet presented ω and ω-*h* values of 0.85 and 0.84, respectively. Thus, it is not appropriate to use a multidimensional scoring procedure in this cognitive facet.

Finally, we examined the construct validity of the FFMQ in Spanish individuals by exploring relationships between the latent factors and anxiety-depression factors that were measured by the DASS-21. Prior to this analysis, we examined the underlying dimensionality of the DASS-21 using Clark and Watson's (1991) tripartite theory of anxiety and depression. The tripartite model posits that in addition to a general factor of negative affect or general distress, there are specific dimensions of anxiety and depression that can be differentiated. Anxiety's specific component is physiological hyper-arousal, while depression's specific component is low positive affect or anhedonia. We found strong empirical evidence for a substantive common factor in the DASS-21 (negative affect/general distress) from the CFA. There was also evidence of one robust specific factor (depression) and two weak specific factors (anxiety and stress), which consisted of 4 and 3 items with significant factor loadings, respectively. This was not the first time that a bifactor model had been tested in the DASS-21. Similar to other anxiety/depression instruments, such as the Hospital Anxiety and Depression Scale (HADS; Luciano et al., 2014), the DASS-21 has shown a clear bifactor structure in nonclinical adults from the UK (Henry and Crawford, 2005), in North-American undergraduates (Osman et al., 2012), in a large Portuguese community sample (Vasconcelos-Raposo et al., 2013), and currently in Spanish nonclinical adults, which supports the cross-cultural validity of the quadripartite model initially posited by Henry and Crawford (2005). In clinical practice, professionals may use the three DASS-21 sub-scales separately, but should be aware that the DASS-21 factors are a blend of variance common to all factors (distress), and variance specific to each construct. As such, we used the three DASS-21 sub-scales separately in our subsequent construct validity model despite the presence of two weak specific factors. Even when a unidimensional measurement model in an instrument, such as the DASS-21, may be useful for practical purposes (the ω-*h* for the general distress factor was 0.88), it can also be useful to consider complex measurement models (Reise et al., 2013). A complex model allowed us to test the unique association of general and group latent factors of the FFMQ with related external criteria (general distress, depression/anhedonia, anxiety/physiological hyperarousal, and stress/psychological tension) using Desrosiers et al. (2013) model as reference.

Our construct validity analysis with the bifactor model corroborated the finding that global mindfulness predicted depression, stress, and general distress. In contrast, the cognitive facets were not predictors of anxiety-depressive symptom dimensions when they were disentangled from mindfulness. Among the facets, only observing emerged as an independent negative predictor of anxiety and stress. Rather, the integration of mindfulness into the cognitive facets provided support for their unique role as buffers of distress. Specifically, the expected significant link between acting with awareness, non-judgment, and non-reactivity with general distress/negative affectivity was supported (Hypotheses 6–8). Additionally, specific stress was predicted by observing, acting with awareness, and non-reactivity. Finally, anxiety was predicted by observing. These findings support the idea that mindfulness components do not operate homogeneously across symptom clusters of depression and anxiety (Desrosiers et al., 2013). Moreover, our results have clinical implications because they support the potential effectiveness of targeting specific facets of mindfulness when treating problems related to distress.

Cash and Whittingham (2010) explored which FFMQ facets predicted symptoms of depression, anxiety, and stress (using the DASS-42) in a community-based Australian sample that included non-meditators and experienced meditators. Non-judgment and acting with awareness were significant predictors of depression, accounting for 6.9 and 9.1% of variance, respectively. Unexpectedly, none of the facets significantly predicted depression/anhedonia in our study. We believe that mindfulness facets and depression (anhedonia) have shown significant negative correlations in previous work (Cash and Whittingham, 2010), because the presence of a general factor of psychological distress or negative affect had not been disentangled from depression using a bifactor approach. Undoubtedly, one of the main advantages of bifactor models is that they provide conceptual clarity, which allows one to test the unique relationships between the target construct/s and external criteria (Chen et al., 2012).

The following set of limitations and shortcomings should be considered in the interpretation of our findings. First, this study was conducted with cross-sectional data. Therefore, we were not able to test the longitudinal invariance of the FFMQ. As such, although we detected statistically significant latent relationships between the distinct FFMQ facets and distress symptoms, it is not possible to establish causal links between these variables. Second, as in multiple previous studies, the present work is based on a large non-clinical sample of individuals who may have minor psychological problems. It is recommended that the analyses be replicated with clinical samples to compare results. The bifactor model posited here would only be clinically useful if it increases the discovery of significant correlates between mindfulness and measures of anxiety, depression, worry, etc., in individuals with a clinical diagnosis, compared to alternative factor models.

In sum, this is the first study in the mindfulness literature demonstrating that a bifactor model outperforms the most consistent account of the FFMQ factor structure, the correlated 5-factor model plus method effects established by Van Dam et al. (2012). We believe that bifactor modeling is a promising approach for understanding the structure of mindfulness. With some exception, our bifactor model and the reliability coefficients revealed that the FFMQ is characterized by item multidimensionality, which is necessary to capture the subtleties and complexities of the psychological constructs being measured. Factor loadings were moderate for both the general factor (with the exception of observing) and the specific factors, which supports the computation of subscale scores. In contrast, the computation of a total FFMQ score does not seem empirically justified. In line with Baer et al. (2008), we also conclude that the FFMQ factor structure is not invariant across meditative practice, as being observing is responsible for the differences between regular meditators and non-meditators. Finally, FFMQ facets did not demonstrate equally predictive power for anxiety-depression symptom clusters. A significant drawback of the classical construct validity analyses is that they do not provide clear information on the unique relations between the construct/s of interests and the variables used as external criteria. When the total and specific FFMQ factors were disentangled in our construct validity analysis, it was clear that mindfulness was the unique significant predictor of depression and distress symptoms. Once mindfulness was combined with the cognitive facets, several emerged as predictors of distress, which highlights the clinical utility of cultivating these facets in mindfulness-based treatments for problems related to distress.

## Author contributions

JA made substantial contribution to the analysis and to the interpretation of the data, drafted the manuscript, provided final approval of the version to be published, and agree to be accountable for all aspects of the work in ensuring that questions related to the accuracy or integrity of any part of the work are appropriately investigated and resolved. JL made substantial contribution to the conception and to the design of the study, drafted the manuscript, provided final approval of the version to be published, and agree to be accountable for all aspects of the work in ensuring that questions related to the accuracy or integrity of any part of the work are appropriately investigated and resolved. AC contributed to the collection, scoring/interpretation of the data, revised the manuscript critically for important intellectual content, provided final approval of the version to be published, and agree to be accountable for all aspects of the work in ensuring that questions related to the accuracy or integrity of any part of the work are appropriately investigated and resolved. AB helped out in the interpretation of data for the work, revised the manuscript critically for important intellectual content, provided final approval of the version to be published, and agree to be accountable for all aspects of the work in ensuring that questions related to the accuracy or integrity of any part of the work are appropriately investigated and resolved. JS contributed to the collection and interpretation of the data, revised the manuscript critically for important intellectual content, provided final approval of the version to be published, and agree to be accountable for all aspects of the work in ensuring that questions related to the accuracy or integrity of any part of the work are appropriately investigated and resolved. JG contributed to the collection and interpretation of the data, revised the manuscript critically for important intellectual content, provided final approval of the version to be published, and agree to be accountable for all aspects of the work in ensuring that questions related to the accuracy or integrity of any part of the work are appropriately investigated and resolved.

### Conflict of interest statement

The authors declare that the research was conducted in the absence of any commercial or financial relationships that could be construed as a potential conflict of interest.

## Footnotes

^1^Prior to assembling a large dataset from different subsamples, it was important to test whether the subsamples were homogeneous concerning the structure of the FFMQ items represented by the covariances or correlations. A test of the equality of the covariance matrices was computed to evaluate the equality of the FFMQ items before pooling the data from different studies. If the covariance matrices do not differ among the studies then the data may be pooled because it is supported that the data originated from the same population. The three subsamples of the total dataset were tested for heterogeneity and were found to be homogeneous (CFI = 0.951; RMSEA = 0.039). This implies that the different samples describe the same population and can therefore be pooled.

^2^As supplementary analysis, we computed *t* tests for group comparisons (meditators, *n* = 305 vs. non-meditators, *n* = 287) on the FFMQ facets. Results from the *t* tests showed that Observing [*t* = 12.17, *p* < 0.01, meditators = 30.7 (4.4) vs. non-meditators = 25.7 (5.5)], Describing [*t* = 2.97, *p* < 0.01, meditators = 30.6 (5.2) vs. non-meditators = 29.2 (6.0)], Acting with awareness [*t* = 3.28, *p* < 0.01; meditators = 27.5 (5.2) vs. non-meditators = 26.0 (5.7)], non-judgment [*t* = 6.35, *p* < 0.01; meditators = 30.9 (6.3) vs. non-meditators = 27.5 (6.9)], and non-reactivity [*t* = 11.00, *p* < 0.01; meditators = 25.0 (3.9) vs. non-meditators = 21.3 (4.4)].distinguished between participants who were regular meditators and those that were non-meditators. The effect sizes (Cohen's d) were not equally distributed among the FFMQ facets, being large in some cases (Observing = 0.90; Non-reactivity = 0.83) and moderate (Non-judgment = 0.51) or small (Describing = 0.24; Acting with awareness = 0.27) in others.
