# Corrigendum: Bifactor analysis and construct validity of the Five Facet Mindfulness Questionnaire (FFMQ) in non-clinical Spanish samples

**DOI:** 10.3389/fpsyg.2016.00706

**Published:** 2016-05-09

**Authors:** Jaume Aguado, Juan V. Luciano, Ausias Cebolla, Antoni Serrano-Blanco, Joaquim Soler, Javier García-Campayo

**Affiliations:** ^1^Teaching, Research & Innovation Unit, Parc Sanitari Sant Joan de DéuSant Boi de Llobregat, Spain; ^2^Primary Care Prevention and Health Promotion Network (redIAPP)Madrid, Spain; ^3^Department of Basic and Clinical Psychology and Psychobiology, Universitat Jaume I de CastellóCastellón de la Plana, Spain; ^4^Fisiopatología de la Obesidad y la Nutrición (CIBERobn)Madrid, Spain; ^5^Department of Psychiatry, Hospital de la Santa Creu i Sant PauBarcelona, Spain; ^6^Centre for Biomedical Research in Mental Health (CIBERSAM)Madrid, Spain; ^7^Department of Psychiatry, Aragon Institute of Health Sciences, Miguel Servet HospitalZaragoza, Spain

**Keywords:** Five Facet Mindfulness Questionnaire, bifactor model, structural equation modeling, anxiety, depression

Reason for Corrigendum:

In the original article, we have neglected to thank our sponsor Instituto de Salud Carlos III (ISCIII)—Network for Prevention and Health Promotion in Primary Care (redIAPP, RD12/0005/0006 & RD12/0005/0008), co-financed with European Union ERDF funds. The authors apologize for this oversight. This error does not change the scientific conclusions of the article in any way.

## Author contributions

All authors listed, have made substantial, direct and intellectual contribution to the work, and approved it for publication.

### Conflict of Interest Statement

The authors declare that the research was conducted in the absence of any commercial or financial relationships that could be construed as a potential conflict of interest.

